# Future Time Perspective and Locomotion Jointly Predict Anticipatory Pleasure in Adolescence: An Integrative Hierarchical Model

**DOI:** 10.3390/ejihpe15110238

**Published:** 2025-11-19

**Authors:** Stefania Mancone, Alessandra Zanon, Adele Gentile, Giulio Marotta, Francesco Di Siena, Lavinia Falese, Pierluigi Diotaiuti

**Affiliations:** Department of Human Sciences, Society and Health, University of Cassino and Southern Lazio, 03043 Cassino, Italy; s.mancone@unicas.it (S.M.); a.zanon@unicas.it (A.Z.); a.gentile@unicas.it (A.G.); giulio.marotta@unicas.it (G.M.); francesco.disiena@unicas.it (F.D.S.); l.falese@unicas.it (L.F.)

**Keywords:** time perspective, regulatory modes, anticipatory pleasure, adolescence, gender differences, sensation seeking, integrative model, hierarchical regression, value-based choice

## Abstract

Objectives: Grounded in Zimbardo’s Time Perspective theory and Regulatory Mode theory, together with developmental accounts of adolescent prospection and value-based choice, this study tests a unified model in which Locomotion (primary) and Future time perspective (secondary) jointly predict Anticipatory Pleasure in adolescence, while considering Assessment, gender, age, and sensation seeking. The goal is to understand how adolescents’ temporal orientation and self-regulation contribute to their motivational and hedonic functioning. Methods: A total of 1540 adolescents (aged 14–19 years) completed validated self-report measures assessing time perspective, regulatory mode (assessment and locomotion), anticipatory and consummatory pleasure, and sensation seeking. Gender differences were examined with independent-samples *t*-tests, while associations among variables were tested using Pearson correlations and hierarchical regression analyses. Results: Female adolescents reported significantly higher levels of future orientation and anticipatory pleasure, while males showed greater sensation seeking. Future time perspective and locomotion were positively correlated with anticipatory pleasure. In the regression analysis, locomotion emerged as the strongest predictor of anticipatory pleasure, followed by future orientation. Sensation seeking was not a significant predictor. Conclusions: The findings underscore the importance of future-oriented thinking and action-driven self-regulation in sustaining adolescents’ capacity to anticipate and derive motivation from future experiences. Gender-based motivational pathways are also highlighted, suggesting the need for differentiated developmental interventions. The study provides new insights into the interplay between time-based cognition and motivational dynamics during adolescence.

## 1. Introduction

Time perspective is a psychological framework that integrates cognitive, emotional, and motivational processes to structure individuals’ understanding of their past, present, and future (Zimbardo & Boyd, 2014; Mello & Worrell, 2014; Kairys & Liniauskaite, 2015; Germano & Brenlla, 2021). It becomes especially salient in adolescence, a pivotal phase of identity formation and self-regulatory change, when youths begin to link past experiences, current choices, and imagined futures into a coherent temporal self that supports stable identity and goal pursuit (Nuttin, 2014; Molinari et al., 2016; Syed & McLean, 2016; Wittmann et al., 2015; McKay et al., 2018). In this period, the temporal dimension denotes the subjective organization of experience across past-present-future, shaping how adolescents prioritize and emotionally engage with different time frames in daily life; the growing capacity to reflect on the past, regulate the present, and project into the future marks the emergence of a “time of the self,” in which time is no longer a mere backdrop but a personal axis around which choices, aspirations, and identity revolve (Kooij et al., 2018; Mello et al., 2022; Shirai & Higata, 2016; S. Liu et al., 2023; Tanrikulu & Mouratidis, 2023; Hipson et al., 2021).

Anchored in Regulatory Mode Theory (Assessment, Locomotion) and in developmental models of adolescent prospection and value-based decision-making, we examine how time-based cognition and action-focused regulation converge on anticipatory pleasure as a motivational outcome. Within this framework, motivation is both shaped by and a driver of time perspective. Its strength and direction vary with goal proximity, personal history, social reinforcement, and developmental context (Pfeifer & Berkman, 2018; Zhu & Burrow, 2023; Simons et al., 2004). Adolescents who envision achievable, personally meaningful future goals tend to show higher motivation and perseverance, provided that they can mobilize effective self-regulation, the capacity to assess, plan, and move toward goals (Wilt & Revelle, 2023; Mucha et al., 2020; Barelli et al., 2022). Autobiographical memory supports this trajectory by enabling recall of events that consolidate the evolving self-concept; selective retrieval can strengthen continuity and autonomy and, through retrieval-induced forgetting (RIF), may privilege memories that align with desired self-views (Q. Liu et al., 2024; Glynn et al., 2022; Lam et al., 2022). At the same time, contemporary sociocultural conditions (academic demands, social media, extracurricular pressures) often impose externally paced, fragmented time, reducing opportunities for self-reflection and autonomous goal setting and making it harder to weave a cohesive narrative linking past, present, and future (Krogh & Madsen, 2024; Carey et al., 2023; Russo-Netzer & Shoshani, 2020; Fomina et al., 2020).

A future-oriented time perspective is generally associated with greater well-being, academic success, and self-regulatory capacity, as it enhances purpose and direction in everyday life, whereas present-focused orientations, especially those driven by hedonism or fatalism, tend to correlate with impulsivity and weaker commitment to long-term goals (Mello et al., 2022; Solomontos-Kountouri & Hatzittofi, 2016; Zimbardo & Boyd, 2014). In terms of regulatory processes, Kruglanski et al. (2018) distinguish two complementary modes of self-regulation: Assessment, involving critical evaluation of goals and strategies, and Locomotion, involving efficient movement from intention to action. Adolescents high in locomotion more readily translate future-oriented thinking into goal-directed behavior, while those strong in assessment are better equipped to plan, reflect, and adjust adaptively (Kruglanski et al., 2018).

A related and underexplored variable in this domain is anticipatory pleasure, the motivational excitement associated with envisioning future positive events, which captures the “wanting” component (as distinct from consummatory “liking”) and reflects the ability to derive motivation from imagined rewards (Gard et al., 2006). Higher anticipatory pleasure may help adolescents sustain effort, tolerate delay, and persist toward long-term goals (Aghevli et al., 2003; Stratta et al., 2011; Du & Lyu, 2021). Sensation seeking, the tendency to pursue novel and intense experiences, can interact with time perspective and self-regulation to shape risk behaviors; while often linked to impulsivity and present hedonism, it may also indicate exploratory drive depending on how it is channeled (Baird et al., 2021; Atherton, 2020; Zuckerman, 1971; Romer et al., 2010; Sulimani-Aidan & Melkman, 2022). Finally, gender differences add nuance to these dynamics: males typically report higher sensation seeking and present orientation, whereas females often show greater future orientation and anticipatory pleasure (Fieulaine & Martinez, 2011; Hao et al., 2024).

### 1.1. Gap and Contribution

Prior work has typically examined time perspective and self-regulation in parallel or in dyads (e.g., Future orientation with academic outcomes; regulatory modes with goal pursuit), whereas anticipatory pleasure has been addressed mostly as a hedonic trait or in clinical contexts, seldom jointly modeled with time perspective and regulatory modes in community adolescents (Zimbardo & Boyd, 2014; Mello et al., 2022; Solomontos-Kountouri & Hatzittofi, 2016; Gard et al., 2006; Stratta et al., 2011). This has left three gaps: limited tests of how Future orientation (cognitive scaffold) and Locomotion (regulatory momentum) jointly account for adolescents’ anticipatory pleasure; scarce evidence on whether this linkage holds while controlling Assessment and covariates (sex, age, sensation seeking) (Kruglanski et al., 2018; Zuckerman, 1971; Manna et al., 2013); and inconsistent attention to gender differences within an integrated framework (Fieulaine & Martinez, 2011, pp. 39–40; Hao et al., 2024). Our study addresses these gaps by integrating the three constructs in one model and by testing whether Locomotion (primary) and Future (secondary) predict anticipatory pleasure, over and above Assessment and individual differences, in a large school-based sample. This design clarifies whether future-based cognition and action-focused regulation converge on the motivational “wanting” component that energizes goal pursuit in adolescence (Zimbardo & Boyd, 2014; Kruglanski et al., 2018; Stratta et al., 2011). Our theoretical contribution is to articulate and test a unified developmental pathway (Future → Locomotion → Anticipatory Pleasure) demonstrating that future-oriented cognition (scaffold) and action initiation (momentum) jointly support anticipatory “wanting” in adolescence within one hierarchical model.

### 1.2. Integrative Theoretical Model

**Figure 1:**
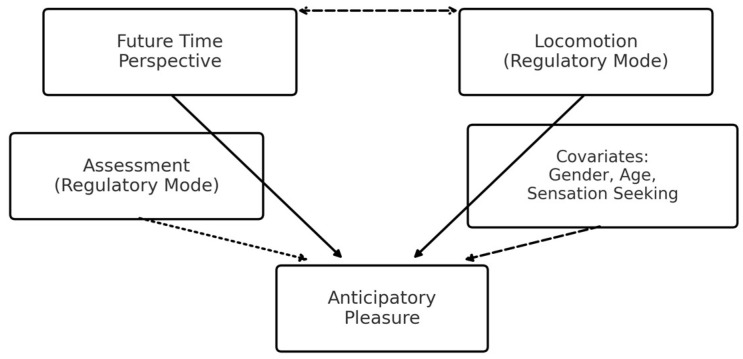
Conceptual model of the hypothesized relationships. Note. Solid arrows = primary hypotheses; dotted = weaker expected association; dashed = covariates.

We propose a developmentally grounded mechanism that coherently integrates Zimbardo’s time perspective and Regulatory Mode traditions: Future provides the cognitive scaffold for goal pursuit (prospective value representation, reduced temporal discounting), Locomotion supplies the regulatory momentum that translates that prospective value into action readiness, and Anticipatory Pleasure indexes the motivational “wanting” energized by this sequence; Assessment operates chiefly at an evaluative layer and is expected to show weaker direct links to anticipatory motivation once Future and Locomotion are considered (Zimbardo & Boyd, 2014; Kruglanski et al., 2018; Stratta et al., 2011; D’Alessio et al., 2003). This integration is adolescent-salient because prospection and identity-linked goal setting strengthen, self-regulatory systems consolidate, and reward sensitivity is heightened during this period (Pfeifer & Berkman, 2018; Romer et al., 2010). Accordingly, we test whether Locomotion (primary) and Future (secondary) jointly account for Anticipatory Pleasure, over and above Assessment and individual differences (sex, age, sensation seeking), clarifying how temporal cognition and action initiation converge on motivated behavior in everyday school contexts. Figure 1 depicts the hypothesized relationships among variables: Future Time Perspective and Locomotion are primary predictors of Anticipatory Pleasure in adolescence; Assessment is included as a control with a weaker direct link; and Gender, Age, and Sensation Seeking enter as covariates. A dashed double-headed arrow denotes the expected correlation between Future and Locomotion.

### 1.3. Objectives and Hypotheses

The present study aims to test whether Locomotion (primary) and Future Time Perspective (secondary) jointly predict Anticipatory Pleasure in adolescence, over and above Assessment, gender, age, and sensation seeking, as depicted in Figure 1.

**H1 (primary).**  *Locomotion → Anticipatory Pleasure (positive). Rationale: in Regulatory Mode Theory, locomotion reflects action initiation and sustained goal pursuit; by translating intentions into progress it energizes prospective “wanting,” i.e., anticipatory pleasure* (Kruglanski et al., 2018; Gard et al., 2006; Stratta et al., 2011).

**H2 (secondary).**  *Future Time Perspective → Anticipatory Pleasure (positive). Rationale: future orientation provides a cognitive scaffold for value representation and delayed gratification, supporting motivated anticipation of rewards* (Zimbardo & Boyd, 2014; Gard et al., 2006; Stratta et al., 2011; D’Alessio et al., 2003).

**H3 (control).**  *Assessment → Anticipatory Pleasure (weaker/non-significant once H1-H2 are modeled). Rationale: Assessment is chiefly evaluative/comparative; when Future and Locomotion are considered, its direct link to anticipatory motivation should attenuate* (Kruglanski et al., 2018).

**H4 (covariate expectation).**  *Sensation Seeking shows little or negative association with Anticipatory Pleasure when H1-H2 are included. Rationale: Sensation seeking indexes present-focused stimulation rather than future-based motivational anticipation* (Zuckerman, 1971; Manna et al., 2013).

**H5 (group differences).**  *Gender differences: males higher in sensation seeking/present-focused tendencies; females higher in future orientation and anticipatory pleasure. Rationale: developmental evidence indicates small but reliable sex-linked patterns in prospection and hedonic-motivational profiles during adolescence* (Pfeifer & Berkman, 2018; Romer et al., 2010; Fieulaine & Martinez, 2011; Hao et al., 2024).

## 2. Materials and Methods

### 2.1. Sample

The study enrolled *n* = 1540 adolescents aged 14–19 years. The sample included 535 males (34.7%) and 1005 females (65.3%). Participants were recruited via random sampling within two high schools, supplemented by friendship-network outreach to broaden sociodemographic diversity. Data collection took place during regular school hours under classroom supervision. Inclusion criteria were current enrollment in grades corresponding to ages 14–19 and adequate language proficiency for questionnaire completion; exclusion criteria were self-reported neurological conditions or ongoing intensive psychiatric treatment. Parental consent and adolescent assent were obtained according to institutional ethical guidelines.

### 2.2. Procedure

The study was conducted in a school setting, with the consent of both the school administrations and the students’ guardians. To ensure a smooth data collection process, researchers visited the participating schools and administered the assessment protocol during scheduled class hours. This approach minimized disruptions to the students’ daily routines and provided a familiar environment for responding to the psychometric scales. Participation was voluntary, and students were informed of their right to withdraw at any time without consequence.

Before data collection, researchers provided a brief orientation to the students, explaining the study’s objectives and reassuring them of the confidentiality of their responses. The orientation highlighted the importance of honest and reflective responses, as the study aimed to understand personal perspectives on time orientation, self-regulation, and pleasure anticipation. This briefing was designed to reduce social desirability bias and ensure that students felt comfortable and motivated to participate. Each participant was given a structured questionnaire packet containing: a demographic questionnaire, which collected information on age, gender, family background, and other relevant socio-demographic factors; four psychometric scales: Zimbardo Time Perspective Inventory (ZTPI) (Zimbardo & Boyd, 2014; D’Alessio et al., 2003); Regulatory Mode Scale (Assessment, Locomotion) (Kruglanski et al., 2018; Pierro et al., 2006); Sensation Seeking Scale—Form V (SSS-V) (Zuckerman, 1971; Manna et al., 2013); and the Anticipatory/Consummatory Pleasure Scale (APS/TEPS) (Gard et al., 2006; Stratta et al., 2011). These scales were presented in a consistent order to control for potential order effects.

With reference to sociodemographic information, we collected the following: parental education and employment, Family Affluence Scale (FAS-III) as a proxy of socioeconomic status (SES), migration background (student and parental country of birth), and living arrangement (two-parent vs. other). School characteristics included school track (lyceum vs. technical) and grade level. Variables were coded as follows: parental education (0 = lower secondary or less; 1 = upper secondary; 2 = tertiary), employment (0 = unemployed/other; 1 = employed), FAS-III standardized and split into quartiles, migration background (0 = both parents born in Italy; 1 = ≥1 parent born abroad), living arrangement (0 = other; 1 = two-parent), school track (0 = technical; 1 = lyceum), grade level (I–V°).

The entire data collection process took approximately 45 min per class. Students completed the assessments individually under the supervision of trained research assistants, who were available to clarify any questions about the items or scales without influencing students’ answers. Upon completion, the questionnaires were collected and securely stored for later data entry and analysis. This study was conducted in line with ethical standards set forth by the American Psychological Association (APA) and the Declaration of Helsinki. Participants’ responses were treated with strict confidentiality, and data were anonymized to protect their identities. Consent was obtained from all participants and, in cases of minors, from their parents or legal guardians. The study was approved by the institutional review board (IRB) of the University of Cassino, ensuring adherence to ethical guidelines throughout the research process.

In order to reduce evaluation apprehension and demand effects, the survey was administered anonymously in classrooms by neutral proctors following standardized instructions emphasizing confidentiality and the absence of right/wrong answers. Different instruments used distinct response formats/anchors and included reverse-keyed items, which help disrupt response routines. The briefing explicitly encouraged honest responding and clarified that participation was voluntary and could be discontinued at any time. These steps aimed to minimize common-method bias (CMB) during data collection.

### 2.3. Instruments

The study utilized a structured assessment protocol that included four psychometric scales along with a preliminary section for collecting socio-demographic information. Each tool was selected for its relevance to key variables in understanding time perspective, self-regulation, and pleasure anticipation. The following instruments were administered:

1. The Zimbardo Time Perspective Inventory (ZTPI), originally developed by Zimbardo and Boyd (2014) and validated in Italian by D’Alessio et al. (2003), is a widely used self-report questionnaire designed to measure individuals’ attitudes toward time across five distinct dimensions. Each of these dimensions reflects a unique temporal orientation, shaping how individuals perceive and respond to their past, present, and future. The ZTPI is particularly relevant for assessing how time perspective influences decision-making, goal setting, and motivational tendencies, which are critical during adolescence. Participants respond to 56 items, indicating their level of agreement or disagreement on a Likert scale (ranging from “Strongly Disagree” to “Strongly Agree”). This format allows participants to express the extent to which each time orientation resonates with their personal views and behaviors. The ZTPI measures five specific time perspectives, capturing a broad range of temporal attitudes: (1) Past-Negative. This dimension assesses the tendency to focus on negative past experiences, such as failures, regrets, and trauma. Individuals scoring high in this area often view their past with disappointment or resentment, which can affect their outlook on the present and future. (2) Past-Positive. This dimension reflects a nostalgic, positive orientation towards past experiences. Those with a high Past-Positive score remember the past fondly and draw strength from positive memories, which can foster a stable sense of identity and security. (3) Present-Hedonistic. This scale measures a present-focused, pleasure-oriented perspective. Individuals high in Present-Hedonistic tend to prioritize immediate gratification, seek stimulating experiences, and often engage in spontaneous or risk-taking behaviors, sometimes disregarding future consequences. (4) Present-Fatalistic captures a belief that life events are governed by fate or chance, leading to a sense of powerlessness over personal outcomes. High scores on this scale are often associated with a passive or resigned attitude toward both present circumstances and future planning. (5) The Future dimension evaluates an individual’s orientation towards future goals, aspirations, and the importance of delayed gratification. High scores indicate a focus on long-term planning and the belief that present actions are instrumental in achieving future success and rewards. The ZTPI has shown moderate to strong internal reliability across its five dimensions, with Cronbach’s alphas ranging from 0.60 to 0.72, indicating moderate reliability for this scale within the sample.
2. The Regulatory Mode Scale (RMS), originally developed by Kruglanski et al. (2018) and validated in Italian by Pierro et al. (2006), is a psychometric tool designed to assess individuals’ self-regulatory tendencies, specifically capturing two distinct regulatory modes: Assessment and Locomotion. These regulatory modes represent different approaches to self-regulation, influencing how individuals pursue goals, make decisions, and engage in daily activities. The RMS is especially relevant in studying adolescent behavior as it provides insights into the processes underlying goal-setting, decision-making, and action-taking, all of which are key aspects of development during this life stage. The RMS consists of 24 items, split evenly between the two subscales (12 items for Assessment and 12 for Locomotion). Each item uses a Likert scale, where participants indicate their level of agreement with statements related to their regulatory style (e.g., “I often critique myself to find areas of improvement” for Assessment; “I am constantly on the move from one activity to another” for Locomotion). This format allows respondents to express their regulatory preferences in various situations, capturing the balance between critical reflection and action orientation. The RMS evaluates self-regulation through two independent yet complementary dimensions: (1) Assessment. This mode reflects an evaluative, comparative approach to self-regulation, where individuals critically assess their current status and alternative paths. People with a high Assessment orientation tend to carefully consider all options and potential outcomes before taking action, aiming to make the “right” decision. This mode is associated with perfectionism, critical self-reflection, and a desire to meet high standards. Individuals high in Assessment are meticulous and detail-oriented, often seeking to improve their decisions and actions through continuous comparison and evaluation. (2) Locomotion represents a dynamic, action-oriented approach to self-regulation, where the primary focus is on moving forward and progressing towards goals without delay or distraction. Those high in Locomotion are motivated to transition smoothly from one activity to the next, prioritizing efficiency and momentum. This mode is linked to proactive behavior, adaptability, and a strong focus on goal completion. Locomotion-oriented individuals are typically less preoccupied with perfection and comparison, concentrating instead on making steady progress and achieving tangible outcomes. This instrument demonstrated good reliability in this study, with a Cronbach’s alpha ranging from 0.70 to 0.76.
3. The Sensation Seeking Scale (SSS-V), initially developed by Zuckerman (1971) and adapted in Italian by Manna et al. (2013), is a psychometric instrument designed to measure individual differences in sensation-seeking behavior. Sensation seeking is defined as the need for varied, novel, and complex experiences and the willingness to take physical and social risks to achieve such experiences. Given its relevance to adolescence, a period often marked by increased exploration, risk-taking, and identity formation, the SSS-V is particularly suitable for studying sensation-seeking tendencies in this age group. The SSS-V is a self-administered questionnaire comprising 40 items, with 10 items per subscale. Each item is presented as a forced-choice statement, where participants select between two options that best describe their preferences (e.g., “I would like to try bungee jumping” vs. “I would never want to try bungee jumping”). This format captures individual differences in sensation-seeking tendencies across the four dimensions, reflecting both attraction to high-stimulation environments and the propensity to engage in risk-taking behaviors. The SSS-V assesses sensation seeking across four subscales, each targeting a distinct aspect of the sensation-seeking trait: (1) Thrill and Adventure Seeking (TAS) captures the desire for excitement and novelty through physically challenging and often risky activities, such as extreme sports. Individuals with high TAS scores are inclined to seek high-stimulation experiences that involve physical risks and excitement. (2) Experience Seeking (ES) reflects a preference for new sensory or cognitive experiences, often through unconventional or creative activities. High scorers on the ES subscale are likely to engage in exploratory behaviors, including travel, art, and unconventional lifestyles. (3) Disinhibition (DIS) measures a tendency towards impulsivity and a lack of restraint, especially in social situations. Individuals with high DIS scores often pursue social risks, such as substance use or unrestrained social interactions, to satisfy their need for stimulation. (4) Boredom Susceptibility (BS) assesses a low tolerance for monotony and repetitive tasks. Individuals scoring high in boredom susceptibility are easily frustrated by routine and seek constant changes to avoid feeling unstimulated. SSS-V demonstrated in this study good internal reliability, with Cronbach’s alpha values ranging from 0.60 to 0.80 for the various subscales.
4. The Anticipatory Pleasure Scale (APS), originally developed by Gard et al. (2006) and validated in Italian by Stratta et al. (2011), is a self-report instrument designed to measure the capacity for anticipatory pleasure, or the positive emotions associated with looking forward to future events. Anticipatory pleasure is a crucial component of the broader hedonic experience, distinguishing the motivation and excitement for potential future rewards (wanting) from the satisfaction derived from immediate experiences (liking). This distinction is especially relevant during adolescence, a developmental period characterized by goal formation and future planning. The APS consists of 18 items that participants rate on a Likert scale, ranging from “Strongly Disagree” to “Strongly Agree”. Ten items measure anticipatory pleasure, while the remaining eight items assess consummatory pleasure. This format allows participants to express the degree to which they experience excitement for upcoming events versus satisfaction from present or past experiences, providing a nuanced view of their hedonic capacity. The APS evaluates two primary aspects of pleasure, each reflecting a distinct stage in the hedonic process: (1) Anticipatory Pleasure (Wanting) focuses on the motivational aspect of pleasure, capturing an individual’s excitement and enthusiasm for future positive events. High scores on this dimension indicate a strong capacity to derive joy from envisioning future experiences, which can influence goal-setting and future-oriented planning. (2) Consummatory Pleasure (Liking) assesses pleasure derived from immediate or completed experiences, such as the enjoyment felt when a desired goal is achieved. While anticipatory pleasure (wanting) is linked to motivation for future actions, consummatory pleasure (liking) reflects satisfaction in the present moment. In the current sample, internal consistency was α = 0.81 for the anticipatory subscale and α = 0.76 for the consummatory subscale.

### 2.4. Statistical Analysis

All descriptive, correlations, group tests, and OLS models were run in IBM SPSS 27. The confirmatory factor analyses (CFA), reliability/validity indices (CR, AVE, HTMT), and measurement invariance were estimated in R (lavaan, semTools) with robust maximum likelihood (MLR). Analysis scripts and output are available upon request.

We inspected ranges, outliers, and univariate normality (skewness/kurtosis) for all variables. For the CFA we used FIML (MLR) to handle sporadic missingness. For OLS regressions, we used listwise deletion on the analysis variables; Ns per model and diagnostics are reported too.

We report means/SDs for all scales and subscales. Gender differences were tested with independent-samples *t*-tests (two-tailed), with Levene’s test for homogeneity of variance and Hedges’ g as effect size.

We estimated Pearson correlations among key variables; coefficients and significance are reported. Effect-size interpretation followed conventional benchmarks.

A four-factor CFA (Future, Locomotion, Assessment, Anticipatory Pleasure) was fit with MLR; fit was evaluated by CFI/TLI (≥0.90–0.95), RMSEA (≤0.06–0.08), SRMR (≤0.08). Convergent and discriminant validity were assessed via: internal consistency (Cronbach’s α, McDonald’s ω), Composite Reliability (CR), Average Variance Extracted (AVE), Fornell–Larcker (√AVE on the diagonal), and HTMT. Summary indices are in Tables S1 and S2; item-level standardized loadings and residuals are in Tables S3 and S4. Sensation Seeking (SSS-V) was treated as an observed composite in structural analyses; subscale reliabilities are reported in Table S1. Primary models: hierarchical OLS predicting Anticipatory Pleasure. We tested our hypotheses with a three-block hierarchical regression (DV: Anticipatory Pleasure): Block 1: Assessment + covariates (sex, age, sensation seeking); Block 2: + Locomotion (primary predictor); Block 3: + Future (secondary predictor). We report standardized β, *p*, R^2^, and ΔR^2^/ΔF for block-wise improvement.

We examined linearity, independence (Durbin-Watson), homoscedasticity (residuals vs. fitted), approximate normality (Q-Q plots, skewness/kurtosis), and multicollinearity (VIF < 10). Diagnostic summaries and residual distribution indices are provided in Table S8; results were robust to HC3 standard errors.

We replicated results with: an APS bifactor model (anticipatory vs. consummatory) and a parceling solution for ZTPI-Future; model fit and conclusions on CR/AVE/HTMT remained stable.

Configural, metric, and scalar gender invariance were tested for the four-factor measurement model; invariance was supported using ΔCFI ≤ 0.010 and ΔRMSEA ≤ 0.015 criteria.

Reporting conventions: two-tailed tests; *p*s are reported in APA style. Effect sizes for correlations and *t*-tests follow standard guidelines; regression coefficients are standardized β. All thresholds are treated as guidelines, interpreted alongside model diagnostics and theory.

We estimated a latent SEM (MLR) with Future, Locomotion, Assessment, and Anticipatory Pleasure as factors defined by their CFA indicators. Anticipatory Pleasure was the endogenous latent outcome. Future predicted Locomotion and both predicted Anticipatory Pleasure, yielding the indirect effect Future → Locomotion → Anticipatory Pleasure. Assessment, sex, age, sensation seeking (SSS-V), SES (FAS-III quartiles), school track, grade level, and school fixed effects were included as exogenous covariates with direct paths to the outcome (and to Locomotion where theoretically warranted). Future ↔ Locomotion latent covariance was freely estimated.

We tested gender moderation via multi-group SEM (male vs. female) under established invariance (configural/metric/scalar supported). SES moderation was probed via latent-observed interactions (centered SES × Locomotion; SES × Future) using orthogonalized product terms. Fit criteria matched the CFA (CFI/TLI, RMSEA, SRMR). Indirect effects used bias-corrected bootstrap CIs (5000 draws).

## 3. Results

### 3.1. Descriptive Statistics

#### Sample Characteristics

The analytic sample comprised 1540 adolescents (males = 535; 34.7%, females = 1005; 65.3%), aged 14–19 years. To facilitate interpretation, age-band proportions were: 14–15 years: 32.0% (*n* = 493), 16–17 years: 46.0% (*n* = 708), 18–19 years: 22.0% (*n* = 339). The chosen age window captures a developmental period in which time perspective and anticipatory pleasure are particularly salient for identity formation and future planning. Participants were drawn through random sampling within two high schools and a complementary friendship-network outreach to increase sociodemographic heterogeneity; the distribution across recruitment channels is summarized in Table 1.

Table 2 below summarizes the mean and standard deviation for each scale and subscale, highlighting the distribution of scores across the sample (*n* = 1540).

An independent samples *t*-test was conducted to assess gender differences across the main study variables. The following Table 3 summarizes the significant findings.

Females scored significantly higher than males on Future Orientation t(1538) = −2.082, *p* = 0.03. Males scored higher in Present Hedonistic orientation t(1538) = 2.082, *p* = 0.02. Females demonstrated higher scores for anticipatory pleasure t(1538) = −2.480, *p* = 0.01. Males scored higher on thrill and adventure seeking within sensation seeking t(1538) = 2.310, *p* = 0.02.

Table 4 below presents the Pearson correlation analyses conducted to examine relationships between the main variables, highlighting significant findings.

As expected, Future and Locomotion were positively associated with Anticipatory Pleasure, whereas Assessment showed a smaller, weaker link. Future and Locomotion were moderately intercorrelated, consistent with the idea that future-oriented cognition tends to co-occur with action-focused regulation. Present-focused tendencies showed a different profile: Present-Hedonistic related weakly to Anticipatory Pleasure, while sensation-seeking subdimensions displayed small negative associations with both anticipatory and consummatory pleasure. Taken together, the pattern supports a Future → Locomotion → Anticipatory Pleasure pathway, with Assessment playing a chiefly evaluative role. For exact coefficients and significance levels, see Table 4.

### 3.2. Reliability, Convergent and Discriminant Validity

Composite reliability and convergent/discriminant validity are detailed in Table S1 (α, ω, CR, AVE) and Table S2 (Fornell–Larcker; HTMT). Item-level evidence is reported in Tables S3 and S4 (standardized loadings and residuals). As robustness checks, we replicated the results with an APS bifactor specification and a parceling solution for ZTPI–Future (Tables S5 and S6); conclusions on CR/AVE/HTMT did not change. Measurement invariance by gender (configural/metric/scalar) held for the four-factor model (Table S7). Residual diagnostics for the OLS models are provided in Table S8.

### 3.3. Anticipatory Pleasure: Hierarchical Prediction from Regulatory Modes and Time Perspective

We tested a three-block hierarchical OLS model with Anticipatory Pleasure as the dependent variable. Q–Q plots and skewness/kurtosis of standardized residuals indicated approximate normality (Table S8). Multicollinearity was low (all VIFs < 2.0). Reversing Block order (Future before Locomotion) did not change significance patterns.

In Block 1 we entered Assessment and covariates (sex, age, sensation seeking). In Block 2 we added Locomotion (primary predictor). In Block 3 we added Future time perspective (secondary predictor).

Block 1 (controls + Assessment): the initial model accounted for a modest share of variance in Anticipatory Pleasure (R^2^ = 0.07, Table 5). Assessment showed a small/weak association (β = 0.06, *p* = 0.034), while covariates had limited contributions: sex (females > males; β = 0.08, *p* = 0.002), age (β = −0.03, *p* = 0.181), and sensation seeking (β = 0.02, *p* = 0.411) were negligible to small in magnitude (Table 5).

Block 2 (+Locomotion): adding Locomotion produced a significant increase in explained variance (ΔR^2^ = 0.12, ΔF = 141.8, *p* < 0.001), and Locomotion emerged as a positive, unique predictor of Anticipatory Pleasure (β = 0.28, *p* < 0.001). With Locomotion in the model, Assessment attenuated and was non-significant/weak (β = 0.03, *p* = 0.222); sensation seeking remained non-significant (β = 0.02, *p* = 0.362) (Table 5 and Table 6).

Block 3 (+Future): entering Future yielded a further, smaller but significant gain (ΔR^2^ = 0.02, ΔF = 27.9, *p* < 0.001). Future was a positive, unique predictor over and above Locomotion and controls (β = 0.11, *p* < 0.001). In the final model, Locomotion retained the largest standardized coefficient (β = 0.26, *p* < 0.001), followed by Future (β = 0.11, *p* < 0.001); Assessment remained weak/non-significant (β = 0.02, *p* = 0.311). Sex differences were small (females slightly higher; β = 0.06, *p* = 0.012); age and sensation seeking were non-significant (βs ≈ −0.02/0.01, *p*s > 0.28) (Table 5).

In line with our hypotheses, Locomotion retained the largest unique association with Anticipatory Pleasure, while Future added a smaller but significant increment in explained variance over and above Assessment and covariates.

### 3.4. Structural Model (SEM): Mediation and Moderation with Simultaneous Controls

The latent SEM showed acceptable fit, χ^2^(248) = 845.6, CFI = 0.950, TLI = 0.943, RMSEA = 0.047, SRMR = 0.045. Standardized paths indicated that Locomotion → Anticipatory Pleasure was the strongest effect (β = 0.42, *p* < 0.001), while Future → Locomotion was moderate (β =.52, *p* < 0.001). The direct path Future → Anticipatory Pleasure was small but positive (β = 0.10, *p* = 0.004). Assessment had a weak/non-significant direct link to Anticipatory Pleasure once Future and Locomotion were included (β = 0.06, *p* = 0.071). Among controls, female sex showed a small positive association (β = 0.07, *p* = 0.018); age, sensation seeking, SES (FAS), school track, and grade were negligible (|β| ≤ 0.06, *p*s > 0.10).

In the SEM, the indirect effect of Future via Locomotion on Anticipatory Pleasure was significant (β indirect = 0.22, 95% CI (0.16;.29)), supporting the hypothesized mechanism (Table S9). Multi-group comparisons indicated no meaningful gender differences in the structural paths (all ΔCFI ≤ 0.005; ΔRMSEA ≤ 0.002 when constraining Locomotion → Anticipatory and Future → Locomotion), consistent with invariant effects across males and females (Table S10). SES moderation was also unsupported: the SES × Locomotion and SES × Future interactions were non-significant (|β| ≤ 0.04, *p*s > 0.20), and the core paths remained unchanged (Table S11). Taken together, these SEM results converge with the hierarchical OLS findings while providing a single, integrated test of the mediational pathway and the moderator/covariate structure.

## 4. Discussion

This study investigated the relationships between time perspective, regulatory modes, anticipatory and consummatory pleasure, and sensation seeking in adolescence, with particular attention to gender differences. The findings provide insight into the motivational and temporal dynamics that shape adolescents’ goal orientation and hedonic functioning (Fajkowska et al., 2023). Rather than aggregating constructs, our findings support a developmentally grounded mechanism in which Future (cognitive scaffold) and Locomotion (regulatory momentum) converge on Anticipatory Pleasure (motivational “wanting”), with Assessment playing a weaker evaluative role (Zimbardo & Boyd, 2014; Kruglanski et al., 2018; Stratta et al., 2011; D’Alessio et al., 2003). This integrated pathway clarifies why these variables are critical in adolescence, a period marked by strengthening prospection, consolidating self-regulation, and heightened reward sensitivity, and constitutes the paper’s primary theoretical contribution beyond sample size and variable combination (Pfeifer & Berkman, 2018; Romer et al., 2010; Steinberg et al., 2009). Within this framework, Future helps represent prospective value and tolerate delay, whereas Locomotion translates that prospective value into action readiness; Assessment, by contrast, operates at a predominantly evaluative layer and shows a weaker direct tie to anticipatory motivation once Future and Locomotion are modeled (Figure 1). The SEM corroborates this structure: the indirect effect Future → Locomotion → Anticipatory is significant, the direct Future → Anticipatory path is small but positive, and Locomotion → Anticipatory remains the strongest link, converging with hierarchical OLS estimates (Table 5, Table 6 and Table S9).

The results revealed significant gender differences across several key variables. Female adolescents reported significantly higher levels of future time perspective and anticipatory pleasure compared to males. This finding aligns with previous research suggesting that females tend to exhibit greater future orientation and emotional introspection during adolescence, potentially due to both biological maturation and sociocultural factors (Hao et al., 2024; Fieulaine & Martinez, 2010; Laghi et al., 2016). In contrast, males scored higher on sensation seeking, consistent with literature showing that adolescent males are more likely to engage in risk-oriented behaviors and prioritize present-focused gratification (Zuckerman, 1971; Romer et al., 2010).

These gender-based patterns suggest distinct motivational pathways in adolescence: while girls may derive motivational energy from future-oriented goals and imagined outcomes, boys may be more driven by present-moment stimulation and novelty. These differences underscore the importance of considering gender as a meaningful moderator in studies on adolescent development and self-regulation.

Correlational analyses showed that a stronger future time perspective was positively associated with anticipatory pleasure and locomotion, indicating that adolescents with a clear orientation toward the future also tend to experience greater motivational engagement and action-oriented regulatory tendencies. This supports the idea that time perspective is not just a cognitive disposition, but also a motivational framework that energizes goal pursuit and prospective thinking (Mello et al., 2022; Nurmi, 2006).

Conversely, present-focused orientation, particularly the hedonistic dimension, was weakly associated with sensation seeking. This suggests that adolescents who prioritize present enjoyment and immediate experiences are more likely to seek intense or novel stimuli, often at the expense of long-term planning. These findings are in line with dual-process models of adolescent behavior, which emphasize the tension between short-term impulse control and long-term goal setting (Steinberg et al., 2009).

Locomotion was positively related to both anticipatory and consummatory pleasure, suggesting that adolescents who are action-oriented and proactive in pursuing goals also tend to experience greater hedonic capacity, both in imagining future rewards and in enjoying present experiences. In contrast, the assessment mode did not show significant associations with pleasure or time perspective, indicating that a reflective, evaluative style may be less directly connected to motivational engagement in adolescence (Vasa et al., 2011; Mancone et al., 2024a, 2024b, 2025a, 2025b).

Beyond statistical significance, it is important to consider the magnitude of the effects observed in this study. For instance, the correlation between future orientation and locomotion (r = 0.44) represents a moderate-to-strong effect according to Cohen’s benchmarks, suggesting that adolescents who are future-oriented are substantially more likely to adopt proactive, goal-directed behaviors. Similarly, the association between assessment and anticipatory pleasure (r = 0.24) falls within the small-to-moderate range, indicating that reflective self-evaluation contributes to anticipatory motivation, although its practical relevance is more limited. Gender differences, while statistically significant, generally showed small effect sizes (Cohen’s d ranging from 0.20 to 0.35), which implies that although patterns differ between males and females, the degree of overlap in distributions remains considerable. These findings highlight that the most practically relevant effects concern the interplay of future orientation and locomotion, rather than gender per se. Thus, interventions aimed at enhancing adolescents’ anticipatory pleasure should prioritize fostering future-oriented thinking and action-driven regulation, as these appear to have the strongest and most meaningful effects on motivational outcomes.

Hierarchical regression analysis clarified the relative contribution of the study variables in predicting anticipatory pleasure. Locomotion emerged as the strongest predictor, followed by future time perspective. These findings highlight the central role of self-regulation through action-oriented processes in sustaining motivational anticipation. Adolescents who are able to initiate and sustain goal-directed behavior are more likely to generate and maintain positive expectations about future outcomes (Kruglanski et al., 2018). Beyond statistical significance, the pattern indicates that Future and Locomotion account for the most practically meaningful variance in anticipatory pleasure (moderate effects by conventional benchmarks), whereas gender differences (though reliable) remain small and should not be over-interpreted (Funder & Ozer, 2019).

The role of future time perspective further emphasizes the cognitive structuring of motivational engagement: the more an adolescent thinks in terms of future goals and rewards, the more likely they are to experience anticipatory emotional activation. Interestingly, sensation seeking did not contribute significantly to the prediction of anticipatory pleasure, suggesting that while both constructs involve motivation, they reflect qualitatively different dynamics, one centered on novelty and immediate arousal, the other on the imaginative projection of reward.

Conceptually, our pattern aligns with value-based choice views of adolescent behavior: future-oriented representations increase the expected value of delayed rewards, while locomotion reduces friction between intention and action, jointly energizing anticipatory pleasure. Boundary conditions likely include contextual opportunity structures (e.g., school track, scaffolding by adults) and regulatory load (e.g., competing demands). In our data, controls for SES (FAS), school track, and grade were negligible, and gender moderation did not change core paths in multi-group SEM, suggesting that the mechanism is relatively general within this school context (Tables S9–S11).

The moderate Future-Locomotion association and the dominance of Locomotion in predicting Anticipatory Pleasure extend prior work that examined these constructs in parallel rather than jointly. Our results suggest that action initiation is the proximal driver of anticipatory “wanting”, while future orientation supplies upstream structure, consistent with theories that separate cognitive prospection from regulatory execution in adolescence. The weak/attenuated role of Assessment once Future/Locomotion are entered implies that evaluative comparison alone is insufficient to energize anticipatory motivation without concomitant momentum.

These results have practical implications for adolescent mental health and education. Encouraging the development of a future-oriented mindset and promoting proactive goal-directed behaviors may enhance adolescents’ capacity to experience anticipatory pleasure, a key factor in motivation, resilience, and well-being.

Taken together, our results support a developmental pathway in which Future time perspective provides the cognitive scaffold for goal pursuit, Locomotion supplies the regulatory momentum that translates prospective reward into action, and anticipatory pleasure indexes the motivational “wanting” that energizes this system. Assessment plays a weaker, chiefly evaluative role. Gender differences, although small, are consistent with this pathway (females: higher Future/anticipatory pleasure; males: higher sensation seeking), suggesting partly overlapping profiles rather than categorical splits. Contextually, these patterns align with international evidence linking future orientation and action initiation to adaptive outcomes, but may vary with school tracking, regional norms, and opportunity structures. For instance, in settings with stronger performance pressures or fewer structured opportunities, the coupling of Future and Locomotion could be either amplified (through goal salience) or attenuated (through resource constraints). Thus, the present Italian school-based findings are likely to generalize to other European samples with similar curricular organization, while cultural moderation (e.g., teacher expectancies, parental scaffolding, digital media ecologies) should be tested explicitly in cross-national designs.

Our findings are broadly convergent with prior work linking future orientation and action-focused self-regulation to adaptive motivation in adolescence and with evidence that Locomotion is the more proximal predictor of goal-directed engagement compared with Assessment (Zimbardo & Boyd, 2014; Mello et al., 2022; Kruglanski et al., 2018; Stratta et al., 2011). The moderate Future-Locomotion correlation is also in line with accounts that couple prospective value representation with regulatory momentum during this developmental period (Pfeifer & Berkman, 2018; Kruglanski et al., 2018).

At the same time, two departures are noteworthy. First, the direct Future → Anticipatory Pleasure path was small once Locomotion entered the model, suggesting that Future contributes mainly indirectly via action initiation. Studies that reported larger direct effects typically did not model Locomotion jointly, have used composite “hedonic” outcomes rather than anticipatory pleasure, or relied on smaller samples without simultaneous controls (Zimbardo & Boyd, 2014; Gard et al., 2006; Stratta et al., 2011). Second, Assessment showed attenuated links to Anticipatory Pleasure after accounting for Future/Locomotion. This contrasts with reports where evaluative styles related to motivation, likely because those models did not partial out the momentum component or used different school/age contexts (Kruglanski et al., 2018).

Taken together, these points specify when adolescent anticipatory “wanting” is most strongly supported, namely, when future-based scaffolding is coupled with regulatory momentum, and constitute the study’s original contribution: a theory-driven, latent-variable test of a Future → Locomotion → Anticipatory Pleasure pathway that remains robust under simultaneous controls, measurement checks, and invariance testing (Table 5, Table 6 and Tables S1, S2, S7 and S9–S11).

### 4.1. Limitations

Despite the relevance of the findings, several limitations must be acknowledged. Random sampling within two schools increases internal validity for those settings; the additional friendship-network outreach improves heterogeneity but does not yield a probability sample of the broader regional student population. The cross-sectional nature of the data prevents causal inference, and all measures were self-reported, which may introduce bias or inflate associations. Accordingly, theoretical claims are limited to associative evidence in school-based samples; longitudinal tests and experimental manipulations of future scaffolding and action initiation are needed to establish directionality and boundary conditions. Future research could adopt a longitudinal design and integrate behavioral tasks or ecological momentary assessment to capture dynamic fluctuations in motivation and pleasure. It would also be valuable to explore how anticipatory pleasure functions in different cultural contexts or in relation to other developmental factors such as academic achievement or emotional regulation.

Our sample included a higher proportion of female participants, which may affect the precision of gender contrasts. While observed gender effects were small, they should be interpreted cautiously, and future studies should balance recruitment or apply stratified/probability sampling (or weights/sensitivity analyses) to enhance comparability across groups.

All instruments employed were developed within Western cultural frameworks and, although validated in Italian, may not fully capture cultural moderators of time perspective or pleasure experience. To assess generalizability beyond Italian school settings, future research should implement cross-cultural comparisons with pre-registered hypotheses, test measurement invariance (configural/metric/scalar) across cultures and genders, and incorporate contextual moderators (e.g., school tracking, parental/teacher scaffolding, media ecologies) to map conditions under which the Future → Locomotion → Anticipatory Pleasure pathway is amplified or attenuated.

The project received publication support from the Italian Ministry of University and Research (MUR; PROBEN-2 Call). This contribution did not fund study design or data collection/analysis and the funder had no role in the design, conduct, interpretation, or decision to submit the manuscript.

### 4.2. Practical Implications

The pattern suggests that interventions are most effective when future-goal scaffolding is explicitly paired with regulatory momentum cues. In school settings, this translates into brief “future imaging” prompts that make upcoming rewards concrete (goal/why), followed by stepwise progress rituals at task onset (how/now: first small step, progress feedback, micro-deadlines). Such routines are low-cost, scalable, and map directly onto the Future → Locomotion → Anticipatory Pleasure pathway. Programs aimed at motivation and well-being in adolescence can combine prospective value framing (future benefits, vivid outcome simulation) with action initiation supports (implementation intentions, checklists, “start” cues). Given that gender differences were small, tailoring may focus more on entry points (e.g., emphasizing future imaging vs. momentum cues) than on different mechanisms.

### 4.3. Recommendations for Future Research

Future work should establish temporal precedence between Future and Locomotion using longitudinal and experimental designs, complement self-report with behavioral and experience-sampling indicators to reduce common-method concerns, and routinely report full psychometric evidence (α, ω, CR, AVE, HTMT) with measurement invariance checks before comparing groups. Studies ought to probe contextual moderators as school track, SES (e.g., FAS), regional/cultural setting, and opportunity structures, via multi-site or cross-national samples, clarifying when the Future → Locomotion → Anticipatory Pleasure pathway is amplified or dampened. Analytically, researchers should estimate latent SEMs that test mediation and moderation simultaneously, use bias-corrected bootstrap confidence intervals for indirect effects, account for clustering (e.g., schools) and apply robust standard errors, while making diagnostics (fit indices, residual checks, multicollinearity) transparent. Pre-registration, open materials/data/code, and planned sensitivity analyses (e.g., parceling alternatives, bifactor checks) will further strengthen cumulative evidence.

## 5. Conclusions

This study integrates time perspective and self-regulation into a coherent account of adolescents’ hedonic motivation. Across a large school-based sample, Locomotion emerged as the strongest unique predictor of anticipatory pleasure, with Future time perspective contributing additional variance, while sensation seeking was not predictive. The pattern of small but consistent gender differences (higher Future and anticipatory pleasure in females; higher sensation seeking in males) suggests distinct, yet partly overlapping, motivational profiles in late adolescence.

These results advance the literature by linking future-oriented cognition with action-focused self-regulation in explaining adolescents’ capacity to derive motivation from imagined outcomes. Conceptually, the findings support models in which future-based planning provides a cognitive scaffold, while locomotion supplies the regulatory momentum needed to translate prospective reward into motivational activation.

At the same time, inferences should be made with caution. The cross-sectional design limits causal claims; self-report measures may inflate shared-method variance; and recruitment, although randomized within two schools and diversified via friendship networks, does not constitute a probability sample of the broader population. Residual diagnostics indicated approximate normality for OLS models and support the robustness of the reported associations, but longitudinal and multi-method evidence is needed to test directionality and mechanisms.

Future research should replicate with longitudinal designs, include behavioral tasks and ecological assessments of motivation, and examine moderators (e.g., academic track, socioeconomic background) to identify subgroups for whom the Future → Locomotion → Anticipatory Pleasure pathway is strongest. Establishing temporal precedence and testing mediational models will clarify whether enhancing future orientation strengthens locomotion, or whether locomotion primarily amplifies the motivational impact of future goals.

The study’s novelty lies in modeling Future and Locomotion together as antecedents of Anticipatory Pleasure within a single hierarchical framework in a large community sample, thereby advancing an integrated developmental account of time-based cognition and action-focused regulation.:

## Figures and Tables

**Table 1:** Demographic characteristics of the sample.

Variable	Category	*n*	%
Sex	Male	535	34.7
	Female	1005	65.3
Age bands	14–15 years	493	32
	16–17 years	708	46
	18–19 years	339	22
Parental education (highest in household)	≤Lower secondary	308	20
	Upper secondary	739	48
	Tertiary	493	32
Parental employment	0 employed	216	14
	≥1 employed	1324	86
Family Affluence (FAS-III quartiles)	Q1 (lowest)	385	25
	Q2	385	25
	Q3	385	25
	Q4 (highest)	385	25
Migration background	Both parents Italy	1263	82
	≥1 parent abroad	277	18
Living arrangement	Two-parent	1201	82
	Other	339	22
School track	Lyceum	847	55
	Technical-vocational	693	45
Grade level	I°	240	15.6
	II°	253	16.4
	III°	355	23.1
	IV°	353	22.9
	V°	339	22
Recruitment	High school A (random sampling)	703	45.7
	High school B (random sampling)	652	42.3
	Friendship networks (outreach extension)	185	12

**Table 2:** Descriptive Statistics for Time Perspective, Self-Regulation, Sensation Seeking, and Anticipatory Pleasure Scales.

Scale	Subscale	Mean (M)	Standard Deviation (SD)
Zimbardo Time Perspective Inventory	Past-Negative	3.10	0.75
	Past-Positive	3.60	0.80
	Present-Hedonistic	2.85	0.70
	Present-Fatalistic	2.60	0.65
	Future	3.55	0.78
Regulatory Mode Scale	Assessment	3.20	0.75
	Locomotion	3.90	0.65
Sensation Seeking Scale	Thrill and Adventure Seeking	4.20	0.70
	Experience Seeking	3.90	0.68
	Disinhibition	4.00	0.72
	Boredom Susceptibility	3.80	0.66
Anticipatory Pleasure Scale	Anticipatory Pleasure (Wanting)	4.50	0.68
	Consummatory Pleasure (Liking)	4.20	0.64

**Table 3:** Gender differences.

Variable	Gender	Mean (M)	SD	t	*p*	Hedges’ g (95% CI)
ZTPI: Future	Female	3.65	0.52	−2.082	0.03	−0.11 (−0.22, −0.01)
	Male	3.45	0.87			
ZTPI: Present-Hedonistic	Male	2.73	0.11	2.082	0.02	0.11 (0.01, 0.22)
	Female	2.44	0.05			
APS: Anticipatory Pleasure	Female	4.71	0.09	−2.480	0.01	−0.13 (−0.24, −0.03)
	Male	4.34	0.11			
SSS-V: Thrill and Adventure Seeking	Male	4.40	0.72	2.310	0.02	0.12 (0.02, +0.23)
	Female	3.98	0.68			

Note. Means are reported with standard deviations (SD).

**Table 4:** Bivariate correlations.

Variable	1	2	3	4	5	6	7	8	9	10	11	12	13
1. Past-Negative	1												
2. Past-Positive	−0.12 **	1											
3. Present-Hedonistic	0.15 **	−0.10 **	1										
4. Present-Fatalistic	0.28 **	−0.08 *	0.28 **	1									
5. Future	−0.25 **	0.15 **	−0.20 **	−0.18 **	1								
6. Assessment	0.18 **	0.05	−0.12 **	0.16 **	0.24 **	1							
7. Locomotion	−0.10 **	0.12 **	−0.18 **	−0.15 **	0.44 **	0.30 **	1						
8. Thrill and Adventure Seeking	0.05	−0.07	0.35 **	0.10 **	−0.05	−0.18 **	−0.16 **	1					
9. Experience Seeking	−0.08 *	0.06	0.25 **	−0.05	−0.03	−0.21 **	−0.10 **	0.45 **	1				
10. Disinhibition	0.12 **	−0.10 **	0.40 **	0.18 **	−0.10 **	−0.15 **	−0.12 **	0.50 **	0.38 **	1			
11. Boredom Susceptibility	0.20 **	−0.08 *	0.30 **	0.15 **	−0.12 **	−0.10 **	−0.08 *	0.40 **	0.25 **	0.35 **	1		
12. Anticipatory Pleasure	−0.05	0.18 **	0.15 **	−0.08 *	0.22 **	0.24 **	0.30 **	−0.15 **	−0.10 **	−0.12 **	−0.08 *	1	
13. Consummatory Pleasure	−0.08 *	0.20 **	0.12 **	−0.10 **	0.18 **	0.20 **	0.28 **	−0.12 **	−0.05	−0.10 **	−0.12 **	0.40 **	1

Note. * *p* < 0.05; ** *p* < 0.01.

**Table 5:** Hierarchical regression predicting Anticipatory Pleasure (standardized coefficients).

**Block 1: Assessment + Covariates**				
**Predictor**	**β (std.)**	**SE**	**T**	***p***
Assessment	0.06	0.03	2.12	0.03
Sex (female = 1)	0.08	0.02	3.06	0.002
Age (years)	−0.03	0.02	−1.34	0.181
Sensation seeking	0.02	0.02	0.82	0.411
**Block 2: +Locomotion (Primary Predictor)**				
**Predictor**	**β (std.)**	**SE**	**T**	***p***
Locomotion (primary)	0.28	0.02	11.9	<0.001
Assessment	0.03	0.03	1.22	0.222
Sex (female = 1)	0.07	0.02	2.78	0.006
Age (years)	−0.03	0.02	−1.45	0.149
Sensation seeking	0.02	0.02	0.91	0.362
**Block 3: +Future (Secondary Predictor)**				
**Predictor**	**β (std.)**	**SE**	**T**	***p***
Locomotion (primary)	0.26	0.02	10.78	<0.001
Future (secondary)	0.11	0.02	4.65	<0.001
Assessment	0.02	0.03	1.01	0.311
Sex (female = 1)	0.06	0.02	2.52	0.012
Age (years)	−0.02	0.02	−1.06	0.29
Sensation seeking	0.01	0.02	0.57	0.568

Notes. Dependent variable: Anticipatory Pleasure. Sex coded 0 = male, 1 = female. Standardized β and two-tailed *p*-values reported.

**Table 6:** Model fit and block-wise comparisons (hierarchical OLS).

Model	R^2^	Adj. R^2^	ΔR^2^	ΔF	*p*(Δ)
Block 1: Assessment + covariates	0.07	0.07	-	-	-
Block 2: +Locomotion	0.19	0.19	0.12	141.8	<0.001
Block 3: +Future	0.21	0.21	0.02	27.9	<0.001

## Data Availability

The data presented in this study are available on request from the corresponding author.

## Supplementary Materials

The following supporting information can be downloaded at: https://www.mdpi.com/article/10.3390/ejihpe15110238/s1, Table S1: Internal consistency and composite reliability. Table S2: Discriminant validity (Fornell-Larcker & HTMT). Table S3: CFA standardized loadings (λ) by item. Table S4: CFA residual variances (θ) and item R^2^. Table S5: Robustness: APS bifactor model (Anticipatory vs. Consummatory) Fit índices. Table S6: Robustness: Parceling solution for ZTPI–Future (Fit indices). Table S7: Measurement invariance by gender. Table S8: Residual diagnostics for the OLS models. Table S9: SEM (MLR): Model fit and standardized structural paths (mediation). Table S10: SEM moderation by gender (multi-group constraints). Table S11: SEM SES moderation (interactions/sensitivity).
