# Supplementary material for: Future Time Perspective and Locomotion Jointly Predict Anticipatory Pleasure in Adolescence: An Integrative Hierarchical Model
Source: Eur J Investig Health Psychol Educ. 2025 Nov 19;15(11):238. doi: 10.3390/ejihpe15110238 (PMC12651657; doi:10.3390/ejihpe15110238)
Supplement: Supplementary file 1 [file ejihpe-15-00238-s001.zip › ejihpe-3903531-supplementary.pdf]

## Supplementary Material: Tables S1-S11

## List:

**S1** Internal consistency and composite reliability;
**S2** Discriminant validity (Fornell-Larcker & HTMT);

**S3** CFA standardized loadings ($\lambda$) by item;
**S4** CFA residual variances ($\theta$) and item $R^2$;
**S5** Robustness: APS bifactor model (Anticipatory vs. Consummatory) Fit indices;
**S6** Robustness: Parceling solution for ZTPI–Future (Fit indices);

**S7** Measurement invariance by gender;
**S8** Residual diagnostics for the OLS models;
**S9** SEM (MLR): model fit and standardized structural paths (mediation);
**S10** SEM moderation by gender (multi-group constraints);
**S11** SEM SES moderation (interactions/sensitivity).

Table S1. Internal consistency and composite reliability (N = 1540)

| Construct (scale) | **k** | **$\alpha$** | **$\omega$** | **CR** | **AVE** |
|---|---|---|---|---|---|
| ZTPI – Future | 13 | .79 | .81 | .82 | .50 |
| RMS – Locomotion | 12 | .84 | .86 | .88 | .56 |
| RMS – Assessment | 12 | .74 | .76 | .78 | .48 |
| APS/TEPS – Anticipatory | 10 | .82 | .83 | .85 | .52 |

Note. k = number of items, $\alpha$ = Cronbach's $\alpha$, $\omega$ = McDonald's $\omega$, CR = Composite Reliability, AVE = Average Variance Extracted.

Table S2. Discriminant validity (Fornell–Larcker & HTMT)

| | **Future** | **Locomotion** | **Assessment** | **Anticipatory** |
|---|---|---|---|---|
| Future (√AVE = .71) | **.71** | .58 | .41 | .35 |
| Locomotion | .44 | **.75** | .49 | .47 |
| Assessment | .26 | .32 | **.69** | .39 |
| Anticipatory | .22 | .35 | .26 | **.72** |

Lower triangle = latent factor correlations (ϕ). Diagonal = √AVE. Upper triangle = HTMT. All HTMT < .85; in every row/column the diagonal element (√AVE) exceeds off-diagonal correlations, satisfying Fornell–Larcker.

Table S3. CFA standardized loadings (λ) by item

| Factor | Item | λ | SE | z | p |
|---|---|---|---|---|---|
| Future | F1 | .58 | .02 | 29.0 | <.001 |
| | F2 | .62 | .02 | 31.3 | <.001 |
| | F3 | .65 | .02 | 33.0 | <.001 |
| | F4 | .67 | .02 | 33.7 | <.001 |
| | F5 | .71 | .02 | 35.8 | <.001 |
| | F6 | .63 | .02 | 31.7 | <.001 |
| | F7 | .66 | .02 | 32.9 | <.001 |
| | F8 | .54 | .02 | 27.8 | <.001 |
| | F9 | .69 | .02 | 34.7 | <.001 |
| | F10 | .72 | .02 | 36.4 | <.001 |
| | F11 | .61 | .02 | 30.7 | <.001 |
| | F12 | .70 | .02 | 35.4 | <.001 |
| | F13 | .64 | .02 | 32.0 | <.001 |
| Locomotion | L1 | .66 | .02 | 33.1 | <.001 |
| | L2 | .70 | .02 | 35.4 | <.001 |
| | L3 | .73 | .02 | 36.9 | <.001 |
| | L4 | .68 | .02 | 34.0 | <.001 |
| | L5 | .76 | .02 | 38.5 | <.001 |
| | L6 | .81 | .02 | 40.8 | <.001 |
| | L7 | .77 | .02 | 38.9 | <.001 |
| | L8 | .72 | .02 | 36.3 | <.001 |
| | L9 | .60 | .02 | 30.1 | <.001 |
| | L10 | .86 | .02 | 43.1 | <.001 |

Table S3. CFA standardized loadings (λ) by item

| Factor | Item | λ | SE | z | p |
|---|---|---|---|---|---|
| . | L11 | 74 | .02 | 37.5 | <.001 |
| | L12 | .63 | .02 | 31.4 | <.001 |
| Assessment | A1 | .56 | .02 | 28.6 | <.001 |
| | A2 | .61 | .02 | 30.6 | <.001 |
| | A3 | .65 | .02 | 32.8 | <.001 |
| | A4 | .58 | .02 | 29.2 | <.001 |
| | A5 | .74 | .02 | 37.5 | <.001 |
| | A6 | .68 | .02 | 34.2 | <.001 |
| | A7 | .70 | .02 | 35.0 | <.001 |
| | A8 | .54 | .02 | 27.3 | <.001 |
| | A9 | .66 | .02 | 33.0 | <.001 |
| | A10 | .57 | .02 | 28.4 | <.001 |
| | A11 | .63 | .02 | 31.6 | <.001 |
| | A12 | .59 | .02 | 29.7 | <.001 |
| Anticipatory | AP1 | .62 | .02 | 31.3 | <.001 |
| | AP2 | .66 | .02 | 33.4 | <.001 |
| | AP3 | .70 | .02 | 35.2 | <.001 |
| | AP4 | .75 | .02 | 37.7 | <.001 |
| | AP5 | .68 | .02 | 34.0 | <.001 |
| | AP6 | .73 | .02 | 36.9 | <.001 |
| | AP7 | .77 | .02 | 38.9 | <.001 |
| | AP8 | .81 | .02 | 40.6 | <.001 |
| | AP9 | .58 | .02 | 29.0 | <.001 |
| | AP10 | .64 | .02 | 32.0 | <.001 |

Table S3. CFA standardized loadings (λ) by item

| Factor | Item | λ | SE | z | p |
|---|---|---|---|---|---|

Note. (F = ZTPI–Future; L = RMS–Locomotion; A = RMS–Assessment; AP = APS/TEPS–Anticipatory)

Table S4. CFA residual variances (θ) and item $R^2$

| **Factor** | **Item** | **θ (residual variance)** | **$R^2$ (= $\lambda^2$)** |
|---|---|---|---|
| Future | F1 | .66 | .34 |
| | F2 | .62 | .38 |
| | F3 | .58 | .42 |
| | F4 | .55 | .45 |
| | F5 | .50 | .50 |
| | F6 | .60 | .40 |
| | F7 | .57 | .44 |
| | F8 | .71 | .29 |
| | F9 | .52 | .48 |
| | F10 | .48 | .52 |
| | F11 | .63 | .37 |
| | F12 | .51 | .49 |
| | F13 | .59 | .41 |
| Locomotion | L1 | .56 | .44 |
| | L2 | .51 | .49 |
| | L3 | .47 | .53 |
| | L4 | .54 | .46 |
| | L5 | .42 | .58 |
| | L6 | .34 | .66 |
| | L7 | .41 | .59 |
| | L8 | .48 | .52 |
| | L9 | .64 | .36 |
| | L10 | .26 | .74 |

| Table S4. CFA residual variances (θ) and item $R^2$ | | | |
|---|---|---|---|
| **Factor** | **Item** | **θ (residual variance)** | **$R^2$ (= $\lambda^2$)** |
| | L11 | .45 | .55 |
| | L12 | .60 | .40 |
| Assessment | A1 | .69 | .31 |
| | A2 | .63 | .37 |
| | A3 | .58 | .42 |
| | A4 | .66 | .34 |
| | A5 | .45 | .55 |
| | A6 | .54 | .46 |
| | A7 | .51 | .49 |
| | A8 | .71 | .29 |
| | A9 | .56 | .44 |
| | A10 | .68 | .32 |
| | A11 | .60 | .40 |
| | A12 | .65 | .35 |
| Anticipatory | AP1 | .62 | .38 |
| | AP2 | .56 | .44 |
| | AP3 | .51 | .49 |
| | AP4 | .44 | .56 |
| | AP5 | .54 | .46 |
| | AP6 | .47 | .53 |
| | AP7 | .41 | .59 |
| | AP8 | .34 | .66 |
| | AP9 | .66 | .34 |
| | AP10 | .59 | .41 |

Table S4. CFA residual variances (θ) and item $R^2$

| **Factor** | **Item** | **θ (residual variance)** | **$R^2$ (= $\lambda^2$)** |
|---|---|---|---|

Note. Residuals and $R^2$ reflect the standardized solution.

Table S5. Robustness: APS bifactor model (Anticipatory vs. Consummatory) (Fit indices)

| **Model** | **$\chi^2$ (df)** | **CFI** | **TLI** | **RMSEA [90% CI]** | **SRMR** |
|---|---|---|---|---|---|
| M-B1: Bifactor (general pleasure + two group factors) | 774.1 (244) | .956 | .949 | .044 [.041–.047] | .039 |
| M-B1 vs. baseline four-factor | — | .005 | .005 | −.002 | −.002 |

Note. Bifactor yields marginally better fit; loadings on the Anticipatory group factor remain strong; conclusions on CR/AVE/HTMT unchanged.

Table S6. Robustness: Parceling solution for ZTPI–Future (Fit indices)

| **Model** | **$\chi^2$ (df)** | **CFI** | **TLI** | **RMSEA [90% CI]** | **SRMR** |
|---|---|---|---|---|---|
| M-P1: Future parceled (4 parcels) within four-factor CFA | 603.3 (164) | .962 | .956 | .045 [.041–.049] | .038 |
| M-P1 vs. baseline four-factor | — | .011 | .012 | −.001 | −.003 |

Note. Parceling slightly improves fit; structural conclusions and validity indices remain stable.

Table S7. Measurement invariance by gender (four-factor model)

| **Invariance level** | **$\chi^2$ (df)** | **CFI** | **TLI** | **RMSEA** | **SRMR** | **ΔCFI** | **ΔRMSEA** |
|---|---|---|---|---|---|---|---|
| Configural | 1,231.5 (492) | .949 | .941 | .047 | .045 | — | — |
| Metric | 1,279.3 (504) | .947 | .941 | .047 | .047 | −.002 | .000 |
| Scalar | 1,336.7 (520) | .943 | .939 | .048 | .049 | −.004 | .001 |

Notes. Invariance supported (ΔCFI ≤ .010; ΔRMSEA ≤ .015). Latent means comparisons are therefore interpretable across genders.

Table S8. Residual diagnostics for the OLS models

| Model | n | Skewness | Excess Kurtosis | Notes |
|---|---|---|---|---|
| M1: Anticipatory pleasure ~ Locomotion + covariates | 1,528 | 0.021 | −0.048 | Standardized residuals (mean ≈ 0, SD ≈ 1) |
| M2: Anticipatory pleasure ~ Locomotion + Future + covariates | 1,524 | 0.079 | 0.067 | Standardized residuals (mean ≈ 0, SD ≈ 1) |
| M3: Anticipatory pleasure ~ Locomotion + Future + Assessment + covariates | 1,517 | −0.014 | 0.031 | Standardized residuals (mean ≈ 0, SD ≈ 1) |

Note. In the model formulas, "~" reads as "is modeled as a function of." The symbol "≈" denotes approximate equality (e.g., mean ≈ 0, SD ≈ 1 indicates standardized residuals).

Table S9. SEM (MLR): model fit and standardized structural paths (mediation)

| **Outcome** | **Predictor** | **Std. β** | **SE** | **z** | **p** |
|---|---|---|---|---|---|
| Locomotion | Future | **.52** | .03 | 17.3 | <.001 |
| Anticipatory Pleasure | Locomotion | **.42** | .04 | 10.7 | <.001 |
| | Future (direct) | **.10** | .03 | 2.9 | .004 |
| | Assessment | .06 | .03 | 1.8 | .071 |
| | Sex (female = 1) | .07 | .03 | 2.4 | .018 |
| | Age | −.03 | .02 | −1.3 | .184 |
| | Sensation seeking | .02 | .02 | 0.9 | .370 |
| | SES (FAS quartiles) | .03 | .02 | 1.2 | .221 |
| | School track (lyceum = 1) | .04 | .02 | 1.5 | .130 |
| | Grade level | −.02 | .02 | −0.8 | .420 |
| | School FE (B vs A) | .01 | .02 | 0.4 | .688 |

Notes. Indirect effect (bootstrap, 5,000): Future → Locomotion → Anticipatory Pleasure: β =. 22, 95% CI [.16;.29]; Total effect Future → Anticipatory: β = .32, 95% CI [.26;.38]. Model fit: $\chi^2(248)$ = 845.6, CF I = .950, TLI = .943, RMSEA = .047 [.043; –.050], SRMR = .045. Standardized coefficients; covariances among exogenous predictors freely estimated.

Table S10. Gender moderation - Multi-group SEM (male vs. female)

| **Constraint tested** | **$\Delta\chi^2$ ($\Delta$df)** | **ΔCFI** | **ΔRMSEA** | **Verdict** |
|---|---|---|---|---|
| Constrain Future → Locomotion equal | 3.2 (1) | .001 | .000 | Invariant |
| Constrain Locomotion → Anticipatory equal | 5.6 (1) | .003 | .001 | Invariant |
| Constrain Future → Anticipatory equal | 2.8 (1) | .001 | .000 | Invariant |

Note. Structural path constraints maintain acceptable fit ($\Delta$CFI ≤ .005, $\Delta$RMSEA ≤ .002), indicating no substantive gender moderation.

Table S11. SES moderation — Interactions and sensitivity

| Outcome | Predictor / Interaction | Std. β | SE | z | p |
|---|---|---|---|---|---|
| Anticipatory Pleasure | Locomotion | **.41** | .04 | 10.2 | <.001 |
| | Future | **.10** | .03 | 2.9 | .004 |
| | SES × Locomotion | −.02 | .02 | −0.9 | .365 |
| | SES × Future | .01 | .02 | 0.5 | .622 |

Notes. SES coded as FAS-III quartiles (centered). Interactions orthogonalized; results robust to alternative codings (terciles; continuous ridit). Core paths remain stable; interactions non-significant.
